# Complexities of Phenological Shifts for Plant–Pollinator Interactions and Ways Forward

**DOI:** 10.1093/icb/icaf034

**Published:** 2025-05-12

**Authors:** Alessandro Fisogni, Natasha de Manincor, Elena Kaminskaia, Nicole E Rafferty

**Affiliations:** Department of Evolution, Ecology, and Organismal Biology, University of California, Riverside, 900 University Avenue, Riverside, CA 92521, USA; Department of Evolution, Ecology, and Organismal Biology, University of California, Riverside, 900 University Avenue, Riverside, CA 92521, USA; Laboratory of Zoology, Research Institute for Biosciences, University of Mons, Place du Parc 20, 7000 Mons, Belgium; School of BioSciences, The University of Melbourne, Parkville, VIC 3010, Australia; School of BioSciences, The University of Melbourne, Parkville, VIC 3010, Australia

## Abstract

Changing climatic conditions can lead to diminished overlap in the timing of flowering and pollinator foraging, potentially resulting in the weakening or loss of plant–pollinator interactions and reducing the fitness of both partners. However, several complexities of phenological shifts limit our ability to predict their consequences for plant–pollinator mutualisms. First, phenological shifts reflect the responses of individuals but are often summarized at the community, species, or population level, potentially obscuring variation that has important implications for interactions within and between species. Second, metrics of phenological asynchrony in pollination, such as temporal overlap between flowering and pollinator foraging, may not accurately characterize changes in interaction strength or fitness costs and benefits and thus are not true metrics of mismatch. Third, our focus has been on shifts in individual life-history events, such as flowering, rather than entire life cycles, despite the physiological integration of seasonal life-history stages (phenophases) that may be under different selection pressures. We suggest that we can advance our understanding of phenological shifts and their consequences for plants and pollinators by studying individual phenological variation in both partners across natural or experimental environmental gradients, measuring interaction rates and their fitness implications in addition to synchrony or overlap, and taking an integrated life cycle approach that can reveal trade-offs. Together, these approaches can yield temporally explicit fitness landscapes for plant and pollinator phenologies and improve our understanding of the consequences of climate change-induced phenological shifts.

## Introduction

Phenology, the timing of life-history events, both shapes and is shaped by the ecology and evolution of populations (Forrest and Miller-Rushing 2010). As global climate change triggers shifts in phenology, interactions between species may be weakened if temporal overlap is reduced, potentially leading to reduced fitness and population declines (Memmott et al. 2007; Freimuth et al. 2022). For many mutualistic interactions, phenological overlap is critical; partner species, which may differ in life history, trophic level, and interdependency, must co-occur at particular developmental stages, or phenophases to exchange benefits (Rafferty et al. 2015). If warming causes plants to begin flowering before pollinators are active, they can suffer reproductive losses (Kudo and Cooper 2019); conversely if pollinators emerge prior to the onset of flowering, they can starve (Schenk et al. 2018a). Indeed, reduced phenological overlap between plants and insect pollinators has been implicated in interaction loss and local extinctions (Burkle et al. 2013). It is therefore important to understand how phenological shifts will affect mutualistic partners and their interactions, including those that provide key ecological functions and useful ecosystem services, such as pollination (Ollerton 2017).

In the context of plant–pollinator mutualisms, flowering phenology shapes community composition (i.e., species identity) and structure (i.e., interaction frequency), exerting influence on pollinator foraging behavior and, because pollinator fitness is often dependent on floral resources, pollinator population dynamics (Ogilvie and Forrest 2017; Ogilvie et al. 2017). Pollinator phenology shapes plant gene flow and reproductive success and similarly structures plant–pollinator interaction networks (Memmott et al. 2007; Duchenne et al. 2020). If flowering and foraging phenologies do not coincide, species cannot interact, and forbidden links are generated in networks (Olesen et al. 2011; de Manincor et al. 2020). Climate change-induced phenological mismatches are thought to be likely between the different trophic levels represented by flowering plants and insect or vertebrate pollinators due to reliance on different cues (e.g., temperature vs. photoperiod; soil vs. air temperatures) or differing sensitivities to the same cues (Thackeray et al. 2016). In addition, because most pollination mutualisms are symmetrically generalized (Waser et al. 1996), partners may not be under selection to respond similarly to changing climatic conditions, making them more likely to develop asynchronies (Rafferty et al. 2015). Even if partner switching alleviates some of the negative fitness consequences of phenological asynchrony for generalized mutualists, selection and community dynamics will be altered (Brosi and Briggs 2013; Gienapp et al. 2014).

Although researchers have speculated for nearly two decades that species-specific changes in response to warming could disrupt mutualisms by causing phenological mismatches (Memmott et al. 2007; Hegland et al. 2009; Rafferty et al. 2015), we are still unraveling complexities in the diagnosis and prognosis of mismatches for plants and pollinators. Here, we identify three such complexities: (1) capturing mosaics of phenological response within and among populations; (2) relating metrics of phenological asynchrony to fitness; and (3) integrating the consequences of phenological shifts across entire life cycles. We conclude with some suggestions for ways we can tackle these complexities and advance our understanding of how phenological shifts will affect pollination mutualisms in this era of global change.

## Complexity 1: How to capture variation in phenology?

Phenological shifts have been relatively well-documented for flowering plants and pollinators. Indeed, the timing of flowering serves as a key biological indicator of climate change, providing a record of the effects of warming temperatures and altered precipitation regimes (Parmesan and Hanley 2015; Büntgen et al. 2022). Flowering is often triggered by temperature, moisture, and photoperiod cues (Wilczek et al. 2010; Borghi et al. 2019), and warmer spring temperatures (in combination with sufficient chilling) generally drive earlier flowering (Cook et al. 2012). The genetic controls of flowering phenology have been studied in the context of climate change (Wilczek et al. 2010; Satake et al. 2013), and there is evidence for adaptive plasticity in flowering time (Anderson et al. 2012) that could enable rapid adjustments to changing cues and perhaps to changing pollinator phenologies, reducing the likelihood of mismatches (Renner and Zohner 2018). Insect phenology has similarly provided a strong signal of global climate change (Forrest 2016; Abarca and Spahn 2021), particularly in the emergence and flight times of insects such as butterflies and bees that serve as important pollinators (Roy and Sparks 2000; Forister et al. 2018; Duchenne et al. 2020; Stemkovski et al. 2020). For bees, emergence time tends to advance with warmer spring temperatures (Slominski and Burkle 2019; Kehrberger and Holzschuh 2019a), but responses depend on body condition (Schenk et al. 2018b) and on life-history traits, such as voltinism, nesting substrate, and overwintering stage (Forrest 2016; Stemkovski et al. 2020).

**Fig. 1:**
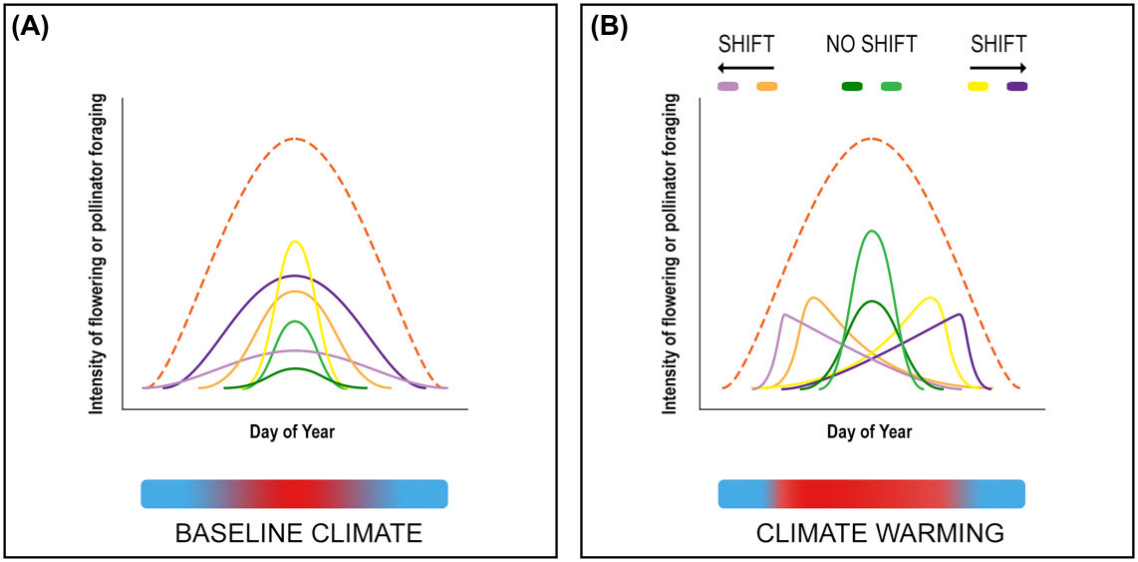
Phenological responses of individual flowering plants or pollinators to changing climatic cues. (A) Individual phenologies (solid lines) under baseline climate conditions. (B) Changing climate (e.g., warming) may alter individual distributions in different ways depending on their sensitivity, modifying their timing, skewness, and breadth. However, divergent responses can be masked when considering cumulative distributions (dashed lines) at the population, species, or community level. The cumulative phenological distributions in (A) and (B) are identical despite being composed of very different individual phenological curves.

Though useful, summary statistics of phenological advancement with climate change fail to capture individual variation in phenology. Most knowledge about phenological shifts comes from community-, species-, or population-level data (Inouye et al. 2019). Although species within communities can respond differently to the same cues, manifested in opposing directions of phenological change or no change, across large spatial and temporal scales it is often only the overall average effect on phenology that is reported and related to climatic cues (e.g., Diez et al. 2012; Rafferty et al. 2020). At the population level, presence/absence or abundance data typically document whether or how many individuals are in a given phenophase (e.g., Fisogni et al. 2022) without identifying and following those individuals over time (Gienapp et al. 2014; Zettlemoyer and DeMarche 2021). In other words, we often do not know how individuals are responding, and we therefore cannot relate phenological change to fitness, limiting our ability to identify the mechanisms underlying variation in survival and reproduction, demography, and ultimately population dynamics. Because identical population-level distributions of flower abundance over time can be produced by multiple, highly dissimilar sets of individual flowering curves (Elzinga et al. 2007; de Keyzer et al. 2017), we cannot decompose population-level data on flower abundance into individual-level data on flowering phenology (Fig. 1). The same holds for pollinator phenological distributions. Thus, although population- and community-level phenological data provide valuable insight into larger-scale spatial patterns (Diez et al. 2012; Rafferty et al. 2020; Fisogni et al. 2022), they can sometimes conceal phenological shifts within populations.

To more fully characterize the phenological changes of flowering plants and pollinators, we need more data on individuals across experimental or natural environmental gradients that encompass variation in the climatic cues that influence phenology (Fig. 1). Ideally, data on individual-level phenological responses can be related to abiotic drivers and used to construct process-based phenological models (Chuine and Régnière 2017) or reaction norms (Inouye et al. 2019) to forecast the effects of further climate change. In addition, data on the phenologies of individuals can reveal local adaptation and plasticity that may be obscured by population-level data. Phenological response diversity of individuals within populations can also be useful in identifying phenological tracking vs. bet-hedging strategies, though confirmation of bet-hedging requires long-term fitness data (Wolkovich and Donahue 2021). Further, phenological response diversity can have very different implications for intra- and interspecific interactions. For example, for a population of plants with a right-skewed flowering season, mate availability and thus the opportunity for sexual vs. asexual reproduction could vary widely depending on whether all individuals have right-skewed flowering periods or the skewness emerges from a larger proportion of individuals flowering earlier in the season (de Keyzer et al. 2017). These different scenarios have similarly variable implications for the temporal distribution of floral resources for pollinators. Additionally, for insect pollinators, within-population variability in life cycle events such as the duration of diapause can influence the timing of emergence, thus influencing the probability of interaction with available floral resources. For these reasons, it is important to understand individual variation in phenology and the drivers of that variation.

Individual phenologies are relatively easy to document for plants via standard field surveys or greenhouse or growth chamber studies, and community science efforts that involve following the phenologies of known individual plants can provide long-term and spatially extensive phenological data (Denny et al. 2014). In addition, digital camera technologies can perform automatic monitoring of temporal changes in plants (i.e., digital repeat photography), which can be used to characterize phenological stages such as flowering at the individual level in the short and long term (Fitchett et al. 2015; Tang et al. 2016). The phenology of vertebrate pollinators, such as birds, can be followed individually via standard field surveys using visual or acoustic observations (McKinney et al. 2012; Robertson et al. 2024), which can benefit from participatory science, or with more advanced radio-frequency and DNA metabarcoding identification (Hazlehurst et al. 2021; English et al. 2022). Such methods allow individual phenologies to be compared with those of host plants, revealing asynchronies and potential mismatches with food resources (Søraker et al. 2022; Robertson et al. 2024). However, individual-level data remain out of reach for most insect pollinators for which individuals cannot be readily distinguished or followed throughout their lifespans in the field or maintained in the laboratory. Although advances in marking and tracking individual insects using a range of approaches based on passive tags, active transmitters, and machine learning increase the feasibility of collecting and processing such data (Mola and Williams 2019; Smith et al. 2022), we are currently limited to sampling individuals of large-bodied, social pollinators, primarily honey bees and bumble bees, with the main goal of determining their movement behavior (Kissling et al. 2014; Mola and Williams 2019). In the near future, it is likely that tracking of individual butterflies over trans-continental distances will become possible, providing detailed data for migratory butterflies such as monarchs (Knight et al. 2019; Green II 2023). Still further technological advances are needed before we can apply these approaches to a wide variety of pollinating insects with different functional traits (e.g., small solitary bees and hover flies), to characterize diverse pollinator communities, and to estimate the period of foraging activity on flowers to link pollinator phenology to pollination potential (Allen-Perkins et al. 2024).

## Complexity 2: How to gauge phenological mismatch?

Phenological synchrony measures the overlap between the temporal distributions of two interacting species; it does not consider the fitness consequences of synchrony for either species (Kharouba and Wolkovich 2020). The empirical definition of phenological mismatch between interacting species requires that fitness is maximized under a particular degree of synchrony, such that a change in synchrony imposes fitness costs. For consumer-resource interactions, this translates to maximum fitness of the consumer when its peak energetic demand is perfectly synchronized with maximum resource availability (Kharouba and Wolkovich 2020). For plant–pollinator mutualisms, a common implicit assumption is that fitness of both partners is maximized when peak pollinator energetic demand (often measured in terms of foraging activity) is synchronized with peak flowering. Although empirical evidence of phenological mismatch between plants and pollinators requires the measurement of fitness costs, this is rarely achieved, as we detail herein. We contend that we need more studies that truly quantify phenological mismatch (i.e., relate variation in synchrony to variation in fitness) to predict the consequences of shifts in phenology for plant–pollinator mutualisms.

Varying degrees of plant–pollinator phenological asynchrony have been identified. Some communities of plants and pollinators appear to maintain phenological overlap due in part to buffering via response diversity (Bartomeus et al. 2011, 2013; Rafferty and Ives 2011; Sevenello et al. 2020). At the same time, the phenologies of some individual species in those communities have become less synchronous (Rafferty and Ives 2012; Bartomeus et al. 2013; Sevenello et al. 2020). Generally, studies have found differing responses and/or sensitivities to abiotic cues among species of flowering plants and insect pollinators which have resulted in (or are likely to generate) asynchronies (Forrest and Thomson 2011; Iler et al. 2013; Kudo and Ida 2013; Kharouba and Vellend 2015; Donoso et al. 2016; Olliff-Yang and Mesler 2018; Kudo and Cooper 2019; Kehrberger and Holzschuh 2019a; but see Cane 2021). Avian pollinators, especially those that migrate, may also experience asynchrony with floral resources as a result of climate change (McKinney et al. 2012; Søraker et al. 2022; Robertson et al. 2024). Few of these studies have measured the fitness consequences of altered synchrony, despite the fact that phenological mismatches are defined by fitness costs (Kharouba and Wolkovich 2020).

Thus, measures of phenological synchrony that are not linked to fitness can suggest mismatches between mutualists but do not offer a complete picture. To characterize phenological synchrony, estimates of phenological overlap that span entire phenophases are more informative than synchrony between single time-point phenological events at distributional extremes, such as flowering onset or pollinator emergence, and are less prone to confounding effects of population size and sampling effort (Miller-Rushing et al. 2008). Measures of synchrony between single events also overlook more complex, discontinuous phenophases for which single values for onset, cessation, and duration cannot easily be extracted; for example, plants in arid and semi-arid ecosystems often have intermittent, multimodal flowering periods (Fisogni et al. 2022). Most informative are estimates that quantify overlap weighted by abundance (Inouye et al. 2019), but even these may not accurately characterize changes in interaction strength or fitness costs and benefits. For instance, the relative amount of overlap between flowering and pollinator nesting seasons may remain constant even as the timeframe of overlap changes. An equivalent amount of temporal overlap between a given plant and pollinator in the first half of the flowering season vs. the second half likely has different fitness implications for both partners. Studies that measure individual fitness under different degrees of phenological overlap (Kuppler et al. 2016) are sorely needed to build a predictive understanding of how climate warming will affect plants and pollinators. At the same time, we need to link plant and pollinator phenology to interaction frequency and net interaction benefit, intermediaries that shape immediate fitness (de Manincor et al. 2023).

Experimental manipulations of phenology offer a powerful approach to determine how phenology affects fitness (Visser and Gienapp 2019). For example, experimentally advanced emergence of the solitary bee *Osmia lignaria* in a natural landscape led to higher fitness, suggesting bees may be under directional selection to emerge earlier (Farzan and Yang 2018). When bees of 3 other species of *Osmia* were placed in flight cages 3 and 6 days before flowers, a forced 6-day asynchrony resulted in reduced survival for all species, whereas 3-day asynchronies had species-specific fitness costs (Schenk et al. 2018a). Similarly, warmer temperatures during *O. ribifloris* development caused delayed emergence and higher mortality (CaraDonna et al. 2018). These studies suggest that developmental temperatures and synchrony with floral resources strongly influence solitary bee fitness. For plants, experimental manipulations of flowering onset in two prairie plant species showed that plants forced to flower early had lower pollination success because they were visited by less-effective pollinators compared to plants flowering at historical times (Rafferty and Ives 2012). Another experimental manipulation of flowering time revealed that an early spring perennial received fewer pollinator visits and had reduced fruit and seed set when flowering was delayed (Gezon et al. 2016). Though limited, these findings suggest mismatches can negatively affect plant reproductive output in the short-term.

**Fig. 2:**
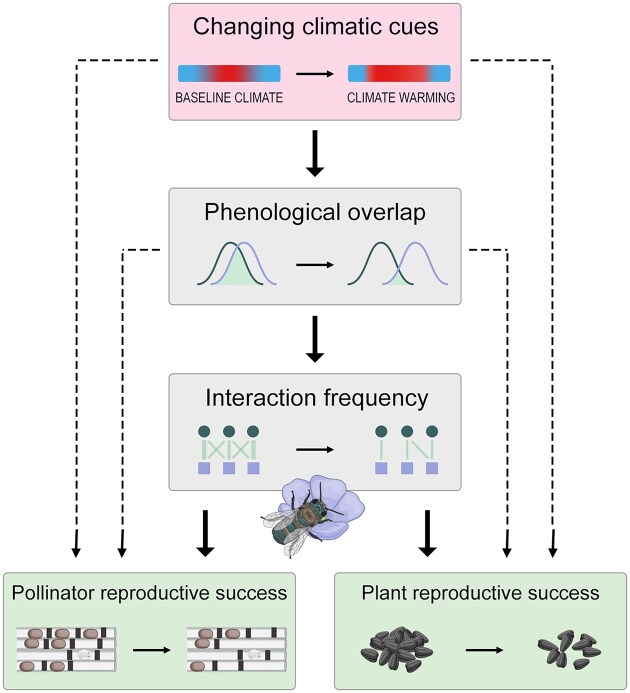
Example of a structural equation model relating variation in climatic cues, such as temperature or precipitation, to plant–pollinator phenological overlap, interaction frequency, and pollinator and plant reproductive success (represented by solitary bee nesting cells with offspring in the prepupal and pupal (cocoon) stages and seed set, respectively). Combined with experimental manipulations of climatic cues that expand the range of variation in flowering and foraging times and phenological overlap, these pathways of direct and indirect effects (solid and dashed arrows, respectively) can be used to study how plant–pollinator mutualisms will be affected by climate change-induced phenological shifts.

Moving forward, if we can relate metrics of plant–pollinator phenological overlap to interaction frequency and then to plant and pollinator reproductive success, we can start to understand the pathways that matter most for each partner (Fig. 2). Combined with experimental manipulations of climatic cues and/or phenology that expand the range of phenotypic variation, we can use these pathways of direct and indirect effects to better understand various scenarios of climate change and how plant–pollinator mutualisms will respond. The key information to be gained from this approach is in the relationship between varying degrees and relative time frames of phenological overlap and immediate fitness metrics. Although a common conceptual model of a phenological match is perfect synchrony, such models have been most thoroughly developed for consumer-resource interactions (Kharouba and Wolkovich 2020), rather than for mutualisms. It is possible that the optimal time frames of overlap differ for plants vs. pollinators; pollinators may benefit from initiating reproduction and foraging closer to peak flowering rather than onset, whereas plants may benefit from initiating flowering after most pollinators have emerged. In other words, optimal overlap for service-resource mutualisms like pollination may arise from some degree of asynchrony that reflects these trade offs and maintains the net benefits of reciprocal exploitation (Kehrberger and Holzschuh 2019b). To achieve insight into what constitutes a phenological mismatch for plant–pollinator mutualisms, temporally explicit metrics of overlap could be used in structural equation models that examine the direct and indirect influence of key variables to connect the dots between phenological responses and proximate fitness.

## Complexity 3: How to integrate shifts across phenophases?

For both plants and pollinators, mismatches may result from shifts in multiple phenological components, such as onset, peak, and end of flowering and foraging (CaraDonna et al. 2014; Stemkovski et al. 2020). Some evidence indicates these components shift independently within plant species (CaraDonna et al. 2014), whereas other work has demonstrated that shifts in onset, peak, and cessation can be correlated within species of plants and bees (Pearse et al. 2017; Stemkovski et al. 2020; but see Iler et al. 2021). The latter suggests that shifts in onset have predictable effects on the timing of later components. Thus, warming that causes mismatches in flowering onset and bee emergence may cause similar mismatches in peak flowering and peak visitation.

However, our focus has been on shifts in individual life-history events, such as flowering or emergence, rather than entire life cycles, despite recognition that downstream phenophases are likely to shift with climate change and experience different selection pressures (Yang and Rudolf 2010). Even between consecutive plant phenophases, such as flowering and fruiting, we have limited understanding of how tightly integrated the timing of these life-history events are (Sandor et al. 2021). Much of the full life cycle data we have comes from studies that address how abiotic factors, such as photoperiod or temperature, affect survival to a particular stage (e.g., Bosch and Kemp 2003), instead of explicitly exploring the knock-on effects of phenological shifts across the life cycle. Exceptions on the plant side include a study that asked whether earlier phenophases, such as leafout, constrain later ones, such as fruiting, across 25 tree species (Ettinger et al. 2018). On the pollinator side, a study on solitary bee response to manipulations of season length found that *Osmia* that experienced an earlier spring had higher pre-emergence mortality and shorter life spans (Slominski and Burkle 2019). Of promise are recent vital rate models that infer phenological abundance distributions of bee populations by linking transitions from unobserved life stages (e.g., pupae overwintering underground) to observed stages (e.g., adults foraging), culminating in senescence (Stemkovski et al. 2024).

**Fig. 3:**
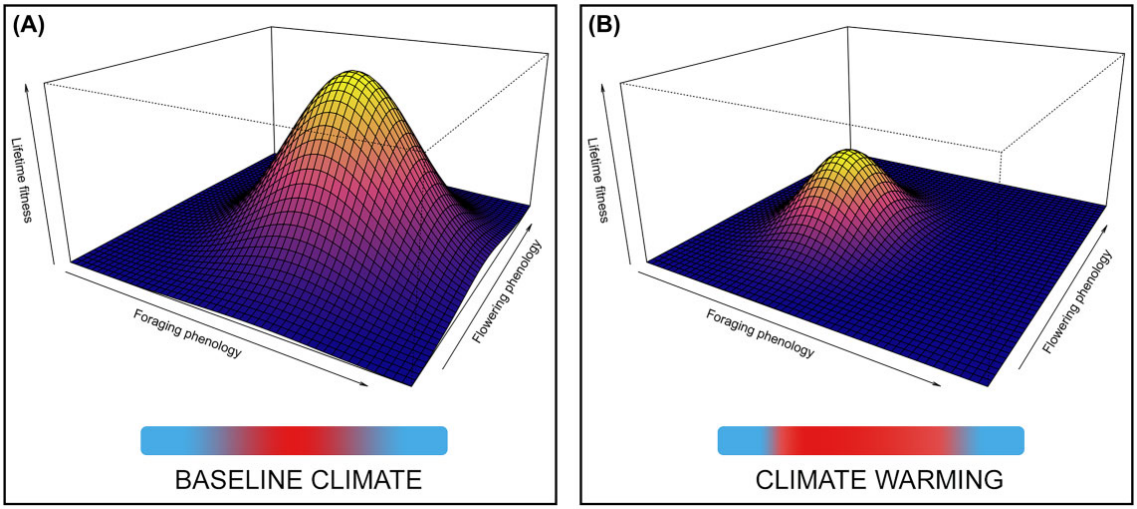
Example of fitness surfaces estimating lifetime fitness probabilities for a focal plant or pollinator species under (A) baseline and (B) changing climate conditions. Studies that iteratively allow sliding windows of overlap between flowering and foraging within experimental populations and then measure the downstream phenological and fitness implications for both partners as they complete their life cycles can reveal fitness peaks corresponding to optimal phenological phenotypes.

We suggest that approaches that explicitly relate plant and pollinator phenologies throughout development to their interactions and to lifetime fitness can provide novel insight into mismatches (Fig. 3), as advocated by Yang and Rudolf (2010) for species interactions more broadly. This type of integrated life cycle approach can reveal trade-offs between phenological responses in different life-history stages (Yang and Rudolf 2010) that could provide insight into why, in addition to reliance on multiple environmental cues that cancel each other out (Cook et al. 2012), some phenophases of some species show no net change in response to changing climatic cues. Though challenging, by mapping trade-offs and constraints across plant and pollinator life cycles that arise from different windows of interaction (Yang and Rudolf 2010), we can gain a more complete understanding of how phenological shifts will play out, at least for short-lived plants and pollinators. Studies that iterate sliding windows of overlap between flowering and foraging in experimental populations and then measure the phenological and fitness implications for both partners as they complete their life cycles could reveal fitness peaks corresponding to optimal windows for each (Fig. 3). These optimal windows, representing optimal phenological phenotypes, could be compared to actual windows of overlap to better diagnose phenological mismatch. If these types of studies were repeated under different experimental treatments, such as warming or drought, different optimal windows of overlap might emerge, suggesting how peak fitness and the associated phenological phenotypes are affected by climate change. Finally, similar studies for multiple species pairs of plants and pollinators could produce community-level fitness landscapes (Stroud et al. 2023) and novel insight into how well-matched we should expect plant and pollinator phenologies to be (Visser and Both 2005; Elzinga et al. 2007).

## Conclusions

In this perspective, we have highlighted three complexities related to how changes in phenology will affect plant–pollinator interactions. Although we have focused on plant–pollinator mutualisms, much of our outlook may be relevant for other species interactions. Greater integration of the concept of phenological mismatch between studies of mutualistic and antagonistic interactions would be valuable, given individuals may experience a spectrum of interaction types during their life cycles. We know that interactions can modify the direct effects of climate change on species (Forrest and Chisholm 2017; Rafferty et al. 2019), and, in the context of phenological synchrony between interacting species, selection on one species may be shaped by the responses of the other. Even under homogeneous environmental change and identical rates of phenological response in interacting species, selection on consumer phenology will invariably occur (Gienapp et al. 2014). Further, conservation of individual species often requires understanding and conservation of their interactions (Heinen et al. 2020). To reduce the complexities we have identified, we point to approaches that can yield temporally explicit fitness landscapes for plant and pollinator phenologies and improve our understanding of the consequences of climate change-induced phenological shifts. Under the current set of wide-ranging threats to pollination services (Goulson et al. 2015), integrative, eco-evolutionary studies can offer much-needed insight into the adaptive capacities of plants and pollinators.

## Author contributions

All authors contributed to the ideas and writing of the manuscript. N.E.R. led the writing of the first draft, with substantial input from A.F. and N.dM. All authors contributed to Fig. design, editing of the manuscript, and to revisions.

## Data Availability

This paper does not use data.

## Notes

From the symposium “Pollinator-plant interactions in a changing landscape: embracing integrative approaches across scales” presented at the annual meeting of the Society for Integrative and Comparative Biology, January 3-7th, 2025.
